# Supplementary material for: Mutations in PCYT2 disrupt etherlipid biosynthesis and cause a complex hereditary spastic paraplegia
Source: Brain. 2019 Oct 22;142(11):3382–97. doi: 10.1093/brain/awz291 (PMC6821184; doi:10.1093/brain/awz291)
Supplement: awz291_Supplementary_Data [file awz291_supplementary_data.zip › awz291-Suppl_data/Supplementary_Data4.zip!/Supplementary information 4 - plasma lipidomics.zip!/Plasma lipidomics/Control vs Patient1/Statistical plots and data/LPE species/Volcano plot_alternative.pdf]

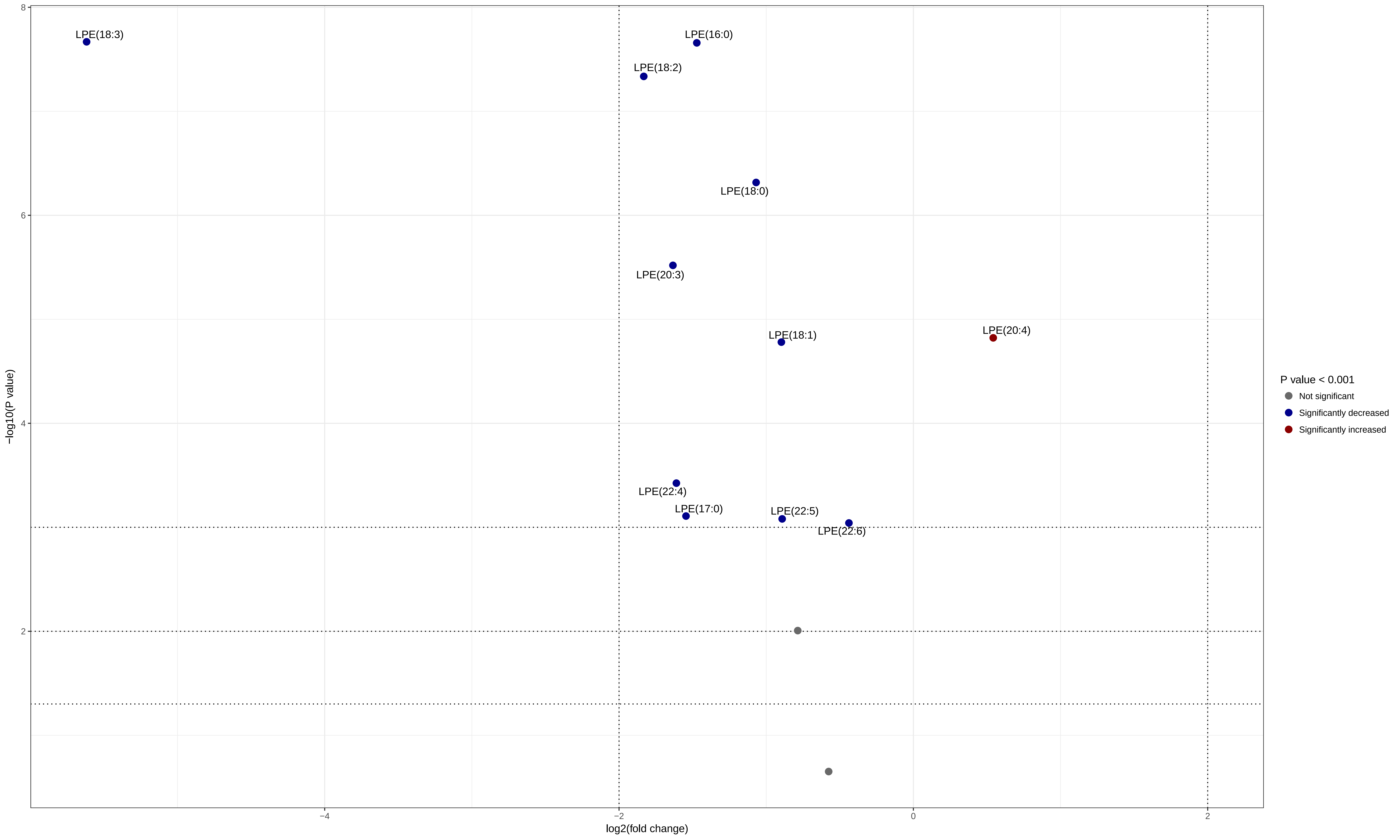
LPE(18:3)
LPE(16:0)
LPE(18:2)
LPE(18:0)
LPE(20:3)
LPE(18:1)
LPE(20:4)
LPE(22:4)
LPE(17:0)
LPE(22:5)
LPE(22:6)
−log10(P value)
log2(fold change)
8
6
4
2
−4
−2
0
2
P value < 0.001
Not significant
Significantly decreased
Significantly increased
